# (*E*)-2-[4-(Dimethyl­amino)styr­yl]-1-methyl­quinolinium iodide sesquihydrate

**DOI:** 10.1107/S1600536808010465

**Published:** 2008-04-18

**Authors:** Suchada Chantrapromma, Thawanrat Kobkeatthawin, Kullapa Chanawanno, Chatchanok Karalai, Hoong-Kun Fun

**Affiliations:** a Department of Chemistry, Faculty of Science, Prince of Songkla University, Hat-Yai, Songkhla 90112, Thailand; b X-ray Crystallography Unit, School of Physics, Universiti Sains Malaysia, 11800 USM, Penang, Malaysia

## Abstract

In the title compound, C_20_H_21_N_2_^+.^I^−^·1.5H_2_O, the cation exists in the *E* configuration and is not planar. The dihedral angle between the quinolinium and dimethyl­amino­phenyl rings is 9.26 (6)°. The O atom of one of the solvent water mol­ecules lies on a twofold rotation axis. In the crystal structure, the cations form one-dimensional zigzag chains along the [001] direction. The cations are linked to water mol­ecules and iodide ions through weak C—H⋯O and C—H⋯I inter­actions, respectively. Water mol­ecules and iodide ions form O—H⋯O and O—H⋯I hydrogen bonds, which stabilize the crystal structure. A C—H⋯π inter­action is also present.

## Related literature

For bond lengths, see: Allen *et al.* (1987 ▶). For background to non-linear optical (NLO) materials research, see: Chia *et al.* (1995 ▶); Marder *et al.* (1994 ▶); Otero *et al.* (2002 ▶); Pan *et al.* (1996 ▶). For related structures, see for example: Chantrapromma *et al.* (2006 ▶, 2007*a* ▶,*b* ▶,*c* ▶,*d* ▶); Dittrich *et al.* (2003 ▶); Jindawong *et al.* (2005 ▶); Kobkeatthawin *et al.* (2008 ▶); Nogi *et al.* (2000 ▶); Sato *et al.* (1999 ▶); Umezawa *et al.* (2000 ▶).
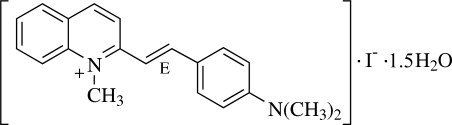


## Experimental

### 

#### Crystal data

C_20_H_21_N_2_^+^·I^−^·1.5H_2_O
*M* *_r_* = 443.31
Monoclinic, 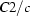

*a* = 20.8997 (4) Å
*b* = 10.5941 (2) Å
*c* = 18.4020 (4) Å
β = 113.047 (1)°
*V* = 3749.24 (13) Å^3^
*Z* = 8
Mo *K*α radiation
μ = 1.72 mm^−1^
*T* = 100.0 (1) K
0.52 × 0.35 × 0.12 mm

#### Data collection

Bruker SMART APEX2 CCD area-detector diffractometer
Absorption correction: multi-scan (*SADABS*; Bruker, 2005 ▶) *T*_min_ = 0.469, *T*_max_ = 0.818
50083 measured reflections
8240 independent reflections
7476 reflections with *I* > 2σ(*I*)
*R*_int_ = 0.032

#### Refinement

*R*[*F* ^2^ > 2σ(*F* ^2^)] = 0.029
*wR*(*F* ^2^) = 0.068
*S* = 1.07
8240 reflections
237 parameters
H atoms treated by a mixture of independent and constrained refinement
Δρ_max_ = 1.58 e Å^−3^
Δρ_min_ = −0.80 e Å^−3^

### 

Data collection: *APEX2* (Bruker, 2005 ▶); cell refinement: *APEX2*; data reduction: *SAINT* (Bruker, 2005 ▶); program(s) used to solve structure: *SHELXTL* (Sheldrick, 2008 ▶); program(s) used to refine structure: *SHELXTL*; molecular graphics: *SHELXTL*; software used to prepare material for publication: *SHELXTL* and *PLATON* (Spek, 2003 ▶).

## Supplementary Material

Crystal structure: contains datablocks global, I. DOI: 10.1107/S1600536808010465/sj2479sup1.cif

Structure factors: contains datablocks I. DOI: 10.1107/S1600536808010465/sj2479Isup2.hkl

Additional supplementary materials:  crystallographic information; 3D view; checkCIF report

## Figures and Tables

**Table 1:** Hydrogen-bond geometry (Å, °)

*D*—H⋯*A*	*D*—H	H⋯*A*	*D*⋯*A*	*D*—H⋯*A*
O1*W*—H1*W*1⋯I1^i^	0.85 (3)	2.74 (3)	3.5832 (16)	172 (3)
O2*W*—H1*W*2⋯O1*W*	0.83 (3)	2.10 (3)	2.9164 (19)	167 (3)
O1*W*—H2*W*1⋯I1^ii^	0.79 (3)	2.94 (3)	3.7267 (16)	174 (3)
C3—H3*A*⋯O2*W*^iii^	0.93	2.60	3.371 (2)	141
C7—H7*A*⋯I1^iv^	0.93	3.04	3.9290 (18)	161
C17—H17*A*⋯I1^ii^	0.93	3.01	3.8784 (14)	157
C2—H2*A*⋯*Cg*1^ii^	0.93	3.02	3.7648 (17)	138

Symmetry codes: (i) 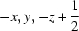; (ii) 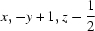; (iii) 
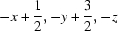
; (iv) 
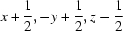
. *Cg*1 is the centroid of the C12–C17 ring.

**Fig. 1.**
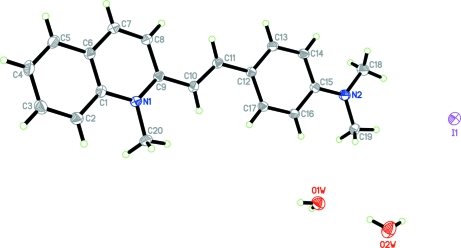
The asymmetric unit of (I) showing 50% probability displacement ellipsoids and the atom-numbering scheme. O2W (symmetry code: -x, y, 1/2 - z) lies on a two-fold rotation axis.

**Fig. 2.**
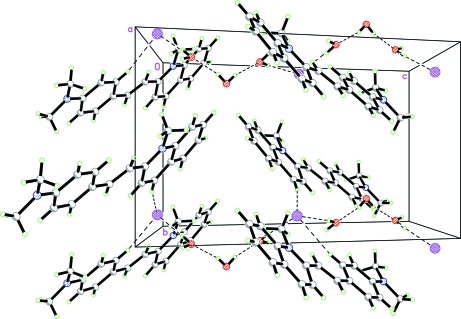
The crystal packing of (I) viewed along the a axis, showing the one-dimensional zigzag chains of the cations running along the c direction. The O—H···O and O—H···I hydrogen bonds and weak C—H···O interactions are drawn as dashed lines.

## supplementary crystallographic information

### Comment

Organic molecules with large π systems have been extensively used in attempts to obtain non-linear optical (NLO) materials (Chia *et al.*, 1995; Dittrich *et al.*, 2003; Marder *et al.*, 1994; Nogi *et al.*, 2000; Otero *et al.*, 2002; Pan *et al.*, 1996; Sato *et al.*, 1999). We have previously synthesized and crystallized several ionic organic salts of quinolinium derivatives which have a conjugate π system to study their non-linear optical properties (Chantrapromma *et al.*, 2006; 2007*a*; 2007*b*; 2007*c*; 2007*d*; Jindawong *et al.*, 2005). Previous investigations by Marder *et al.*, 1994, Pan *et al.*, 1996 and Umezawa *et al.*, 2000 reported that 1-methyl-4-(2-(4-(dimethylamino)phenyl)ethenyl)pyridinium *p*-toluenesulfonate (DAST) is a promising second-order NLO material. Based on this information and our previous investigation (Chantrapromma *et al.*, 2007*c*), we have designed and synthesized the title compound (I) with the replacement of the 3-hydroxy-4-methoxyphenyl ring  in the cation of 2-[(*E*)-(3-hydroxy-4-methoxyphenyl)ethenyl]-1-methylquinolinium iodide monohydrate which showed second-order NLO properties (Chantrapromma *et al.*, 2007*c*) by the 4-dimethylaminophenyl ring and its crystal structure was reported here. However since second-order NLO effects are created only when chromophores are arranged in a non-centrosymmetric manner, the title compound, which crystallized in the centrosymmetric space group *C*2/*c*, does not exhibit any second-order NLO properties.

The asymmetric unit of the title compound consists of one C_20_H_21_N_2_^+^ cation, one I^-^ anion and 1.5 H_2_O molecules. The remaining cell contents are generated by symmetry with the O2W atom (symmetry code: -*x*, *y*, 1/2 - *z*) lying on a two-fold rotation axis. The cation exists in the *E* configuration with respect to the C10═C11 double bond [1.357 (2) Å] and is not planar as indicated by a dihedral angle of 9.26 (6)° between the quinolinium and the dimethylaminophenyl rings. This value is relatively wider than the corresponding angle (3.41 (7)°) reported for the closely related structure of the 4-methoxybenzenesulfonate salt of the same cation (Kobkeatthawin *et al.*, 2008). This may be due to packing effects involving the different counterions. The orientation of the ethenyl unit with respect to the quinolinium and the dimethylaminophenyl rings can be indicated by the torsion angles C8–C9–C10–C11 = 8.5 (2)° and C10–C11–C12–C17 = -1.2 (2)°.  The bond lengths and angles are in normal ranges (Allen *et al.*, 1987) and are comparable to those in  closely related structures (Chantrapromma *et al.*, 2006; 2007*a*; 2007*b*; 2007*c*; Kobkeatthawin *et al.*, 2008).

In the crystal packing (Fig. 2), the cations form one-dimensional zigzag chains along the [0 0 1] direction. Water molecules contribute to an O2W—H1W2···O1W hydrogen bond. The cations are linked to water molecules and iodide ions through weak C—H···O and C—H···I interactions respectively (Table 1). Water molecules and iodide ions are interconnected by O—H···I hydrogen bonds (O1W—H1W1···I1 and O1W—H2W1···I1 symmetry codes: -*x*, *y*, 1/2 - *z* and *x*, 1 - *y*, -1/2 + *z*, respectively). The crystal is further stabilized by O—H···O and O—H···I hydrogen bonds together with weak C—H···O and C—H···I interactions. A C2—H2A···π interaction to the dimethylaminophenyl ring [C12–C17] was also observed:  C2—H2A = 0.93; H2A···*Cg*^i^ = 3.0219; C2—*Cg*_1_^i^ = 3.7648 (17) Å; C2—H2A···*Cg*_1_^i^ = 138°. [*Cg*_1_^i^ is the centroid of the C12–C17 ring (symmetry code: (i): *x*, 1 - *y*, -1/2 + *z*)].

### Experimental

The title compound was synthesized by mixing a 1:1:1 molar ratio solution of 1,2-dimethylquinolinium iodide (2.00 g, 7.01 mmol), dimethylaminobenzaldehyde (1.05 g, 7.01 mmol) and piperidine (0.70 g, 7.01 mmol) in hot methanol (50 ml). The resulting solution was refluxed for 6 h under a nitrogen atmosphere. The resulting solid was filtered off, washed with methanol and recrystallized from methanol to give green crystals. Single crystals of the title compound suitable for *x*-ray structure determination were recrystalized from methanol/ethanol solvent (1:1 *v*/*v*) by slow evaporation of the solvent at room temperature after a few weeks. (Mp. 491–493 K).

### Refinement

Water hydrogen atoms were located in a difference map and refined isotropically. H atoms attached to C were placed in calculated positions with d(C—H) = 0.93 Å, *U*_iso_=1.2*U*_eq_(C) for aromatic and CH, 0.96 Å, *U*_iso_ = 1.5*U*_eq_(C) for CH_3_ atoms. A rotating group model was used for the methyl groups. The highest residual electron density peak is located at 0.57 Å from I1 and the deepest hole is located at 0.46 Å from I1.

### Crystal data

**Table:**

C_20_H_21_N_2_^+^·I^–^·1.5H_2_O	*F*_000_ = 1784
*M**_r_* = 443.31	*D*_x_ = 1.571 Mg m^−^^3^
Monoclinic, *C*2/*c*	Melting point = 491–493 K
Hall symbol: -C 2yc	Mo *K*α radiation λ = 0.71073 Å
*a* = 20.8997 (4) Å	Cell parameters from 8240 reflections
*b* = 10.5941 (2) Å	θ = 2.1–35.0º
*c* = 18.4020 (4) Å	µ = 1.72 mm^−^^1^
β = 113.047 (1)º	*T* = 100.0 (1) K
*V* = 3749.24 (13) Å^3^	Block, green
*Z* = 8	0.52 × 0.35 × 0.12 mm

### Data collection

**Table:**

Bruker SMART APEX2 CCD area-detector diffractometer	8240 independent reflections
Radiation source: fine-focus sealed tube	7476 reflections with *I* > 2σ(*I*)
Monochromator: graphite	*R*_int_ = 0.032
Detector resolution: 8.33 pixels mm^-1^	θ_max_ = 35.0º
*T* = 100.0(1) K	θ_min_ = 2.1º
ω scans	*h* = −33→33
Absorption correction: multi-scan(SADABS; Bruker, 2005)	*k* = −17→15
*T*_min_ = 0.469, *T*_max_ = 0.818	*l* = −29→28
50083 measured reflections	

### Refinement

**Table:**

Refinement on *F*^2^	Secondary atom site location: difference Fourier map
Least-squares matrix: full	Hydrogen site location: inferred from neighbouring sites
*R*[*F*^2^ > 2σ(*F*^2^)] = 0.029	H atoms treated by a mixture of independent and constrained refinement
*wR*(*F*^2^) = 0.068	*w* = 1/[σ^2^(*F*_o_^2^) + (0.0246*P*)^2^ + 7.1959*P*] where *P* = (*F*_o_^2^ + 2*F*_c_^2^)/3
*S* = 1.07	(Δ/σ)_max_ = 0.001
8240 reflections	Δρ_max_ = 1.58 e Å^−^^3^
237 parameters	Δρ_min_ = −0.80 e Å^−^^3^
Primary atom site location: structure-invariant direct methods	Extinction correction: none

### Special details

**Table:**

Experimental. The low-temparture data was collected with the Oxford Cryosystem Cobra low-temperature attachment.
Geometry. All e.s.d.'s (except the e.s.d. in the dihedral angle between two l.s. planes) are estimated using the full covariance matrix. The cell e.s.d.'s are taken into account individually in the estimation of e.s.d.'s in distances, angles and torsion angles; correlations between e.s.d.'s in cell parameters are only used when they are defined by crystal symmetry. An approximate (isotropic) treatment of cell e.s.d.'s is used for estimating e.s.d.'s involving l.s. planes.
Refinement. Refinement of *F*^2^ against ALL reflections. The weighted *R*-factor *wR* and goodness of fit *S* are based on *F*^2^, conventional *R*-factors *R* are based on *F*, with *F* set to zero for negative *F*^2^. The threshold expression of *F*^2^ > σ(*F*^2^) is used only for calculating *R*-factors(gt) *etc*. and is not relevant to the choice of reflections for refinement. *R*-factors based on *F*^2^ are statistically about twice as large as those based on *F*, and *R*- factors based on ALL data will be even larger.

### Fractional atomic coordinates and isotropic or equivalent isotropic displacement parameters (Å^2^)

**Table:**

	*x*	*y*	*z*	*U*_iso_*/*U*_eq_	
I1	0.121463 (5)	0.401312 (10)	0.489877 (6)	0.02205 (3)	
N1	0.34849 (6)	0.45765 (12)	−0.04848 (7)	0.0163 (2)	
N2	0.16088 (7)	0.23257 (14)	0.29101 (8)	0.0219 (2)	
C1	0.39458 (7)	0.47357 (14)	−0.08616 (8)	0.0173 (2)	
C2	0.38630 (8)	0.57284 (16)	−0.13994 (9)	0.0222 (3)	
H2A	0.3488	0.6279	−0.1525	0.027*	
C3	0.43416 (9)	0.58795 (17)	−0.17383 (10)	0.0249 (3)	
H3A	0.4283	0.6533	−0.2096	0.030*	
C4	0.49123 (8)	0.50731 (18)	−0.15566 (9)	0.0244 (3)	
H4A	0.5234	0.5202	−0.1784	0.029*	
C5	0.49965 (8)	0.40906 (16)	−0.10418 (9)	0.0219 (3)	
H5A	0.5371	0.3542	−0.0929	0.026*	
C6	0.45170 (8)	0.39079 (14)	−0.06823 (8)	0.0183 (2)	
C7	0.45974 (8)	0.29187 (16)	−0.01383 (9)	0.0211 (3)	
H7A	0.4961	0.2347	−0.0029	0.025*	
C8	0.41475 (8)	0.27979 (15)	0.02247 (9)	0.0201 (3)	
H8A	0.4206	0.2142	0.0581	0.024*	
C9	0.35853 (7)	0.36612 (14)	0.00684 (8)	0.0163 (2)	
C10	0.31320 (7)	0.35646 (14)	0.04900 (8)	0.0173 (2)	
H10A	0.2735	0.4065	0.0326	0.021*	
C11	0.32564 (7)	0.27790 (14)	0.11134 (8)	0.0170 (2)	
H11A	0.3652	0.2275	0.1265	0.020*	
C12	0.28250 (7)	0.26643 (13)	0.15584 (8)	0.0157 (2)	
C13	0.29994 (7)	0.18026 (14)	0.21874 (9)	0.0185 (2)	
H13A	0.3396	0.1309	0.2309	0.022*	
C14	0.26020 (7)	0.16647 (14)	0.26310 (9)	0.0193 (2)	
H14A	0.2730	0.1075	0.3038	0.023*	
C15	0.20009 (7)	0.24148 (14)	0.24709 (8)	0.0164 (2)	
C16	0.18213 (7)	0.32815 (14)	0.18357 (8)	0.0177 (2)	
H16A	0.1428	0.3783	0.1714	0.021*	
C17	0.22208 (7)	0.33918 (14)	0.13970 (8)	0.0176 (2)	
H17A	0.2088	0.3964	0.0981	0.021*	
C18	0.17497 (9)	0.13464 (18)	0.35064 (10)	0.0262 (3)	
H18A	0.2231	0.1376	0.3857	0.039*	
H18B	0.1646	0.0535	0.3254	0.039*	
H18C	0.1465	0.1481	0.3801	0.039*	
C19	0.09759 (8)	0.30611 (17)	0.27093 (10)	0.0236 (3)	
H19A	0.1078	0.3939	0.2680	0.035*	
H19B	0.0792	0.2943	0.3107	0.035*	
H19C	0.0640	0.2788	0.2208	0.035*	
C20	0.28968 (8)	0.54554 (16)	−0.07022 (10)	0.0223 (3)	
H20A	0.2575	0.5167	−0.0483	0.034*	
H20B	0.2667	0.5494	−0.1267	0.034*	
H20C	0.3064	0.6280	−0.0499	0.034*	
O1W	0.03830 (8)	0.54957 (15)	0.13362 (8)	0.0297 (3)	
O2W	0.0000	0.6809 (2)	0.2500	0.0385 (5)	
H1W2	0.0114 (15)	0.633 (3)	0.2215 (16)	0.044 (8)*	
H1W1	0.0030 (16)	0.508 (3)	0.1033 (17)	0.049 (8)*	
H2W1	0.0580 (16)	0.564 (3)	0.1055 (18)	0.049 (8)*	

### Atomic displacement parameters (Å^2^)

**Table:**

	*U*^11^	*U*^22^	*U*^33^	*U*^12^	*U*^13^	*U*^23^
I1	0.01875 (5)	0.02151 (5)	0.02561 (5)	−0.00453 (3)	0.00840 (4)	−0.00498 (4)
N1	0.0154 (5)	0.0157 (5)	0.0180 (5)	0.0016 (4)	0.0066 (4)	0.0002 (4)
N2	0.0203 (5)	0.0256 (6)	0.0235 (6)	0.0038 (5)	0.0126 (5)	0.0083 (5)
C1	0.0164 (5)	0.0198 (6)	0.0165 (5)	−0.0016 (5)	0.0073 (5)	−0.0020 (5)
C2	0.0190 (6)	0.0242 (7)	0.0236 (6)	0.0016 (5)	0.0086 (5)	0.0041 (5)
C3	0.0233 (7)	0.0289 (8)	0.0229 (7)	−0.0006 (6)	0.0096 (6)	0.0055 (6)
C4	0.0214 (6)	0.0351 (8)	0.0199 (6)	−0.0028 (6)	0.0117 (5)	−0.0010 (6)
C5	0.0180 (6)	0.0282 (7)	0.0205 (6)	0.0022 (5)	0.0088 (5)	−0.0032 (5)
C6	0.0177 (6)	0.0205 (6)	0.0167 (5)	0.0008 (5)	0.0067 (5)	−0.0009 (5)
C7	0.0198 (6)	0.0222 (7)	0.0219 (6)	0.0056 (5)	0.0088 (5)	0.0013 (5)
C8	0.0183 (6)	0.0222 (7)	0.0214 (6)	0.0051 (5)	0.0095 (5)	0.0036 (5)
C9	0.0168 (5)	0.0153 (5)	0.0173 (5)	0.0002 (4)	0.0072 (5)	−0.0001 (4)
C10	0.0182 (6)	0.0172 (6)	0.0185 (6)	−0.0002 (5)	0.0093 (5)	−0.0006 (5)
C11	0.0149 (5)	0.0184 (6)	0.0179 (5)	0.0003 (4)	0.0068 (4)	−0.0001 (5)
C12	0.0149 (5)	0.0160 (5)	0.0162 (5)	−0.0004 (4)	0.0060 (4)	0.0004 (4)
C13	0.0162 (5)	0.0194 (6)	0.0199 (6)	0.0031 (5)	0.0071 (5)	0.0036 (5)
C14	0.0183 (6)	0.0193 (6)	0.0208 (6)	0.0028 (5)	0.0082 (5)	0.0057 (5)
C15	0.0152 (5)	0.0173 (6)	0.0164 (5)	−0.0011 (4)	0.0059 (4)	0.0012 (4)
C16	0.0161 (5)	0.0182 (6)	0.0187 (6)	0.0021 (4)	0.0067 (5)	0.0036 (5)
C17	0.0179 (6)	0.0179 (6)	0.0178 (6)	0.0023 (5)	0.0079 (5)	0.0041 (5)
C18	0.0246 (7)	0.0310 (8)	0.0267 (7)	0.0036 (6)	0.0143 (6)	0.0111 (6)
C19	0.0213 (6)	0.0291 (8)	0.0236 (6)	0.0040 (6)	0.0125 (5)	0.0025 (6)
C20	0.0209 (6)	0.0212 (7)	0.0284 (7)	0.0064 (5)	0.0133 (6)	0.0048 (5)
O1W	0.0271 (6)	0.0340 (7)	0.0266 (6)	0.0012 (5)	0.0092 (5)	0.0014 (5)
O2W	0.0530 (13)	0.0278 (10)	0.0469 (12)	0.000	0.0329 (11)	0.000

### Geometric parameters (Å, °)

**Table:**

N1—C9	1.3614 (19)	C11—C12	1.4411 (19)
N1—C1	1.4001 (18)	C11—H11A	0.9300
N1—C20	1.4672 (19)	C12—C13	1.406 (2)
N2—C15	1.3611 (18)	C12—C17	1.408 (2)
N2—C19	1.453 (2)	C13—C14	1.381 (2)
N2—C18	1.454 (2)	C13—H13A	0.9300
C1—C2	1.408 (2)	C14—C15	1.417 (2)
C1—C6	1.412 (2)	C14—H14A	0.9300
C2—C3	1.380 (2)	C15—C16	1.417 (2)
C2—H2A	0.9300	C16—C17	1.3750 (19)
C3—C4	1.397 (2)	C16—H16A	0.9300
C3—H3A	0.9300	C17—H17A	0.9300
C4—C5	1.372 (2)	C18—H18A	0.9600
C4—H4A	0.9300	C18—H18B	0.9600
C5—C6	1.414 (2)	C18—H18C	0.9600
C5—H5A	0.9300	C19—H19A	0.9600
C6—C7	1.413 (2)	C19—H19B	0.9600
C7—C8	1.356 (2)	C19—H19C	0.9600
C7—H7A	0.9300	C20—H20A	0.9600
C8—C9	1.427 (2)	C20—H20B	0.9600
C8—H8A	0.9300	C20—H20C	0.9600
C9—C10	1.4442 (19)	O1W—H1W1	0.85 (3)
C10—C11	1.357 (2)	O1W—H2W1	0.79 (3)
C10—H10A	0.9300	O2W—H1W2	0.83 (3)
			
C9—N1—C1	121.43 (12)	C12—C11—H11A	117.4
C9—N1—C20	121.57 (12)	C13—C12—C17	116.83 (12)
C1—N1—C20	116.99 (12)	C13—C12—C11	120.29 (13)
C15—N2—C19	120.78 (13)	C17—C12—C11	122.88 (13)
C15—N2—C18	120.43 (13)	C14—C13—C12	122.22 (13)
C19—N2—C18	118.03 (12)	C14—C13—H13A	118.9
N1—C1—C2	121.28 (13)	C12—C13—H13A	118.9
N1—C1—C6	119.48 (13)	C13—C14—C15	120.47 (13)
C2—C1—C6	119.22 (13)	C13—C14—H14A	119.8
C3—C2—C1	119.55 (15)	C15—C14—H14A	119.8
C3—C2—H2A	120.2	N2—C15—C14	121.92 (13)
C1—C2—H2A	120.2	N2—C15—C16	120.54 (13)
C2—C3—C4	121.53 (15)	C14—C15—C16	117.54 (12)
C2—C3—H3A	119.2	C17—C16—C15	120.92 (13)
C4—C3—H3A	119.2	C17—C16—H16A	119.5
C5—C4—C3	119.73 (14)	C15—C16—H16A	119.5
C5—C4—H4A	120.1	C16—C17—C12	122.01 (13)
C3—C4—H4A	120.1	C16—C17—H17A	119.0
C4—C5—C6	120.29 (14)	C12—C17—H17A	119.0
C4—C5—H5A	119.9	N2—C18—H18A	109.5
C6—C5—H5A	119.9	N2—C18—H18B	109.5
C1—C6—C7	118.76 (13)	H18A—C18—H18B	109.5
C1—C6—C5	119.66 (14)	N2—C18—H18C	109.5
C7—C6—C5	121.58 (14)	H18A—C18—H18C	109.5
C8—C7—C6	120.39 (14)	H18B—C18—H18C	109.5
C8—C7—H7A	119.8	N2—C19—H19A	109.5
C6—C7—H7A	119.8	N2—C19—H19B	109.5
C7—C8—C9	121.03 (14)	H19A—C19—H19B	109.5
C7—C8—H8A	119.5	N2—C19—H19C	109.5
C9—C8—H8A	119.5	H19A—C19—H19C	109.5
N1—C9—C8	118.76 (13)	H19B—C19—H19C	109.5
N1—C9—C10	120.71 (13)	N1—C20—H20A	109.5
C8—C9—C10	120.53 (13)	N1—C20—H20B	109.5
C11—C10—C9	123.26 (13)	H20A—C20—H20B	109.5
C11—C10—H10A	118.4	N1—C20—H20C	109.5
C9—C10—H10A	118.4	H20A—C20—H20C	109.5
C10—C11—C12	125.20 (13)	H20B—C20—H20C	109.5
C10—C11—H11A	117.4	H1W1—O1W—H2W1	102 (3)
			
C9—N1—C1—C2	−176.27 (14)	C7—C8—C9—N1	3.4 (2)
C20—N1—C1—C2	2.6 (2)	C7—C8—C9—C10	−176.63 (14)
C9—N1—C1—C6	1.9 (2)	N1—C9—C10—C11	−171.48 (14)
C20—N1—C1—C6	−179.27 (14)	C8—C9—C10—C11	8.5 (2)
N1—C1—C2—C3	177.80 (15)	C9—C10—C11—C12	179.05 (14)
C6—C1—C2—C3	−0.3 (2)	C10—C11—C12—C13	179.02 (14)
C1—C2—C3—C4	−0.4 (3)	C10—C11—C12—C17	−1.2 (2)
C2—C3—C4—C5	1.3 (3)	C17—C12—C13—C14	0.0 (2)
C3—C4—C5—C6	−1.4 (2)	C11—C12—C13—C14	179.79 (14)
N1—C1—C6—C7	1.5 (2)	C12—C13—C14—C15	−1.0 (2)
C2—C1—C6—C7	179.65 (14)	C19—N2—C15—C14	−176.95 (15)
N1—C1—C6—C5	−177.92 (13)	C18—N2—C15—C14	−7.2 (2)
C2—C1—C6—C5	0.2 (2)	C19—N2—C15—C16	3.7 (2)
C4—C5—C6—C1	0.6 (2)	C18—N2—C15—C16	173.45 (15)
C4—C5—C6—C7	−178.79 (15)	C13—C14—C15—N2	−178.22 (15)
C1—C6—C7—C8	−2.3 (2)	C13—C14—C15—C16	1.2 (2)
C5—C6—C7—C8	177.06 (15)	N2—C15—C16—C17	178.95 (15)
C6—C7—C8—C9	−0.1 (2)	C14—C15—C16—C17	−0.5 (2)
C1—N1—C9—C8	−4.2 (2)	C15—C16—C17—C12	−0.5 (2)
C20—N1—C9—C8	176.94 (14)	C13—C12—C17—C16	0.7 (2)
C1—N1—C9—C10	175.76 (13)	C11—C12—C17—C16	−179.05 (14)
C20—N1—C9—C10	−3.1 (2)		

### Hydrogen-bond geometry (Å, °)

**Table:**

*D*—H···*A*	*D*—H	H···*A*	*D*···*A*	*D*—H···*A*
O1W—H1W1···I1^i^	0.85 (3)	2.74 (3)	3.5832 (16)	172 (3)
O2W—H1W2···O1W	0.83 (3)	2.10 (3)	2.9164 (19)	167 (3)
O1W—H2W1···I1^ii^	0.79 (3)	2.94 (3)	3.7267 (16)	174 (3)
C3—H3A···O2W^iii^	0.93	2.60	3.371 (2)	141
C7—H7A···I1^iv^	0.93	3.04	3.9290 (18)	161
C17—H17A···I1^ii^	0.93	3.01	3.8784 (14)	157
C2—H2A···Cg1^ii^	0.93	3.02	3.7648 (17)	138

Symmetry codes: (i) −*x*, *y*, −*z*+1/2;  (ii) *x*, −*y*+1, *z*−1/2; (iii) −*x*+1/2, −*y*+3/2, −*z*; (iv) *x*+1/2, −*y*+1/2, *z*−1/2.
